# Patient Recommendations for the Content and Design of Electronic Returns of Genetic Test Results: Interview Study Among Patients Who Accessed Their Genetic Test Results via the Internet

**DOI:** 10.2196/29706

**Published:** 2022-05-31

**Authors:** Diane M Korngiebel, Kathleen McGlone West

**Affiliations:** 1 University of Washington Seattle, WA United States

**Keywords:** user-centered design, genomic medicine, patient portals, electronic health records, return of results, bioethics, genetics, genetic testing, patient preferences, design, human factors, mobile phone

## Abstract

**Background:**

Genetic test results will be increasingly made available electronically as more patient-facing tools are developed; however, little research has been done that collects data on patient preferences for content and design before creating results templates.

**Objective:**

This study identifies patient preferences for the electronic return of genetic test results, including what considerations should be prioritized for content and design.

**Methods:**

Following user-centered design methods, 59 interviews were conducted by using semistructured protocols. The interviews explored the content and design issues of patient portals that facilitated the return of test results to patients. We interviewed patients who received electronic results for specific types of genetics tests (pharmacogenetic tests, hereditary blood disorder tests, and tests for the risk of heritable cancers) or electronically received any type of genetic or nongenetic test results.

**Results:**

In general, many of participants felt that there always needed to be some clinician involvement in electronic result returns and that electronic coversheets with simple summaries would be helpful for facilitating this. Coversheet summaries could accompany, but not replace, the more detailed report. Participants had specific suggestions for such results summaries, such as only reporting the information that was the most important for patients to understand, including next steps, and doing so by using clear language that is free of medical jargon. Electronic result returns should also include explicit encouragement for patients to contact health care providers about questions. Finally, many participants preferred to manage their care by using their smartphones, particularly in instances when they needed to access health information on the go.

**Conclusions:**

Participants recommended that a patient-friendly front section should accompany the more detailed report and made suggestions for organization, content, and wording. Many used their smartphones regularly to access test results; therefore, health systems and patient portal software vendors should accommodate smartphone app design and web portal design concomitantly when developing platforms for returning results.

## Introduction

Health care systems must provide timely electronic access to genetic test results within the terms of Health Insurance Portability and Accountability Act regulations and meet meaningful use requirements [1]. Genetic test results that are provided in person by a genetics professional allow for appropriate counseling and prompt clinical management decisions, but increasingly more health systems are delivering test results via patient portals that are linked to electronic health records (EHRs). This approach supports the 21st Century Cures Act in facilitating patients’ access to information in their EHRs [2]. Nearly 80% of health care providers use certified EHRs [3], and with that use comes the potential to communicate with patients through EHR-linked patient portals [4]. The expansion of the applications of genetic testing and the increased role of patient-facing health information technology together present an opportunity to design genetic test report content and web-based report templates to respect the information needs and preferences of diverse patients. To ensure a patient- and user-centered approach, patient input is needed to guide the design and content of electronic reporting templates at the outset.

Although there have been robust discussions on the return of genetic results [5-7], including the results of carrier screening [8,9], results for research participants [10-12], and incidental findings [13], and a growing amount of literature studying patient portal usage [14-16], there have been few studies concerning the design aspects for the electronic return of genetic test results via patient portals [17-19]. These issues may be particularly fraught in the context of results that can be sensitive or difficult to understand [20]. Often, results need to be interpreted carefully in terms of patients’ medical and family histories; a negative result, for example, may have a different significance depending on how strongly a patient’s family history suggests the presence of an inherited disorder [21].

Ensuring that portals are tailored to meet patient needs has the potential to not only ensure the appropriate delivery of results but also enable the use of patient portals to encourage appropriate follow-ups [22]. For example, some studies have demonstrated that patients respond to electronic reminders, which are sent through patient portals, to schedule screenings and other preventive services [23]. Although more research is needed, some patient populations may require additional support to use portals [24,25] and understand genetic test results in particular [26]. Patients from underserved populations [27] and those with limited health literacy may need engagement methods that assist them in effective portal and information use [26,28-30]. Some research has suggested that patient preferences for the return of negative genetic test results or normal nongenetic results [31] generally exhibit more openness for impersonal returns (such as electronic returns) than that for returns of results that are positive or abnormal; however, patient preferences vary greatly, and as noted previously, negative genetic results may have nuanced implications [32,33]. Additionally, in general, patients can misinterpret risk [34]. However, patient recipients are interested in participant-driven approaches, such as user-centered design, that consider how results are delivered [35]. As more and more genetic results are returned to patients via electronic portals, more understanding of these design elements is necessary to ensure that patients are able to not only access their medical information but also understand the implications of these results for their families’ and their own health. We sought to identify patient perspectives on design-related issues, such as those regarding the content, formatting, and structure of reports, with the electronic return of clinical genetic test results and other test results to patients.

## Methods

### Participant Recruitment

We identified patients from within the University of Washington (UW) Medicine EHR system who had undergone a genetic test within the 12 months prior to the start of recruitment and had also been active on the UW Medicine patient portal. Participants were invited if they had undergone genetic tests corresponding to 1 of the following 3 levels of concern, as identified by the study team and project advisory board: (1) fraught (ie, positive results for hereditary cancer risk), (2) moderately fraught (ie, blood coagulation genetic risk or α-thalassemia risk), and (3) not fraught (ie, pharmacogenetic or negative results indicating that the patient did not have a pathogenic variant). Pharmacogenetic results were considered to be not fraught, as they only have implications for how a health care provider treats a known condition (eg, selecting the safest and most effective drug or dosage for the patient’s metabolism) rather than for predicting disease risk or indicating the presence of a condition.

Patient information was queried through the Institute for Translational Health Sciences bioinformatics research service, which maximized patient privacy prior to enrollment, as researchers only had access to eligible patient names, contact information, basic demographics, and the types of genetic tests that patients underwent; they did not have access to additional details about patients’ health or reasons for testing. Potential participants were invited by email or phone up to 3 times per person until we reached the stratified sampling goals for test types and data saturation. We prioritized invitations to ensure a broad representation of available demographics (age, gender, race, and ethnicity). Participant demographics (N=59) are summarized in Table 1.

**Table 1:** Participant demographics (N=59).

Characteristics		Value
Age (years), mean (SD)		48.5 (15.3)
**Age (years), n (%)**		
	<30	7 (12)
	30-39	15 (25)
	40-49	8 (14)
	50-59	11 (19)
	60-69	12 (20)
	≥70	6 (10)
**Self-reported gender, n (%)**		
	Female	39 (66)
	Male	20 (34)
**Race and ethnicity, n (%)**		
	African American or Black	4 (7)
	Asian	5 (9)
	American Indian or Alaska Native	0 (0)
	Hispanic or Latinx	5 (9)
	White	43 (73)
	Unreported	2 (3)
**Type of result^a^, n (%)**		
	Fraught	18 (31)
	Moderately fraught	32 (54)
	Not fraught	9 (15)

^a^Fraught results included positive results for cancer risk variants, moderately fraught results included blood coagulation types and α-thalassemia test results, and not fraught results included pharmacogenetic and negative cancer risk variants.

### Ethics Approval

This study was approved by the UW Institutional Review Board (STUDY00005045).

### Data Collection

A semistructured interview guide (Textbox 1) was developed by the study team with guidance from the project advisory board, and it was piloted to ensure its appropriateness for a patient audience. To minimize participant burden, about half of the participants (30/59, 51%) discussed design-related issues based on their experiences with receiving a specific genetic test result through the patient portal, and about half (29/59, 49%) were asked to compare their experiences with receiving a specific genetic test result through the patient portal to their experiences with receiving a nongenetic test result that they identified through the patient portal (eg, cholesterol levels, blood counts, and radiology reports).

In-depth telephone interviews were conducted from May to August 2019, were audio-recorded, and lasted for an average of 35 minutes. Participants were offered a modest gift card for their participation. All interviews were professionally transcribed, and transcripts were deidentified and reviewed for accuracy.

**Selected questions from semistructured interview guide.**

**Questions for genetic and specific nongenetic results**

- “What was it like for you to receive your test result on eCare?”
- “What was good about the experience? What would have improved the experience?”
- “How were you able to interpret (or make sense of) the results?”
- “What, if anything, did you do with the test result that you received? What role, if any, did electronic return play in the usefulness of the test result?”

**Questions for genetic results only**

- “How would you describe your understanding of the results reported in eCare?”
- “What information was included with your *[insert specific genetic test]* result in eCare?”
- “What did you think about the text and visual materials?”
- “Can you give an example of what was communicated clearly?”
- “What could have been communicated more clearly?”
- “Would you have preferred to have more or less information available through eCare?”
- “What information, if any, was provided about next steps?”

### Data Analysis

A short, design-focused coding scheme was developed by 2 qualitative analysts. The first analyst (KMW) coded all transcripts for design-related elements by using Atlas.ti 8 software (Scientific Software Development GmbH). The second analyst (DMK) then performed a directed content analysis to identify specific design element themes within a set of categories derived deductively from the interview guide (eg, suggestions for layout, content, organization, wording, etc). Themes were identified deductively and were based on topics that participants raised during the interviews. For example, a summary coversheet was not mentioned in the interview guide, but as more participants suggested the functions of a summary, this topic was explored by interviewers more explicitly and comprised an inductively derived theme within the *content* category.

## Results

### Summary of Results

We interviewed 59 UW Medicine patient users of the electronic patient portal in Washington State. The sample was predominantly White (43/59, 73%) and female (39/59, 66%) and represented a wide range of ages (mean 48.5; range 26-78 years; Table 1). The key domains discussed covered how the electronic result returns would appear to the users (design) and considerations for what is contained in the result returns (content).

### Domain 1: Design of Electronic Result Returns

#### Design Recommendation 1: Include a Simple Summary Coversheet in the Electronic Report That Summarizes the More Detailed Report

Participants generally felt that a summary would be helpful and that less information would be preferred. They offered some analogies for how this summary might appear:

...Say this was something like cancer risk. And [the coversheet summary] would comment on this is likely an inherited risk, therefore [it] could impact your family. And I wouldn't go anything beyond that. And then [beyond] that could be up for the discuss[ion] with your provider.

Participant #25

One participant compared the electronic summary to the abstract of a manuscript:

You'd lay it out basically like an abstract for a research paper. You tested positive or negative against this whatever, then you go to the next part, because of this result, this will affect your treatment in this way. After that, next probable steps to take will be a couple of these things based on what your doctors have said.

Participant #34

Another participant referred to the coversheet that one receives during car maintenance as a valuable framework:

I kind of want it to be like when I go to my car dealership and I get my car serviced and they give you like, this is where your car stats are at, your battery's great, your tire was a little low. We adjusted this.... I want a summary page…I also want an opening cover that says here's your test results. This is what the results mean. This is what the markers mean. This is how it applies to you, what it means for you. This is my area [of] concern or not concern. This is the next step I think you should take.

Participant #43

#### Design Recommendation 2: Include the Electronic Summary Coversheet to Supplement, but Not Replace, the Detailed Clinical Report

Although many participants wanted a brief, patient-friendly summary, some participants also valued the clinical report because it serves as a matter of record—one that is available on the internet—of the test details that might be difficult to remember from a conversation. They may wish to use these details to further explore their specific variants after a clinic visit. One participant said:

In genetic testing there are so many variations,...but you didn't bring paper to write that down...exactly what that was or what that means. So then to have it in writing so I can see “oh it's this gene mutation” with all the numbers and the letters that go along with it.... So I can have that documented and then if I want to do more research online I can do that...copy that I can look at.... Details, once again, confirmation in my head that I heard correctly.

Participant #29

Other participants appreciated the value that a detailed electronic report could have for other clinicians, viewing it as a part of their medical history that would be relevant to future care:

I think it's good to have them [the detailed results] through [the patient portal], because I know having the records on there, other doctors can access [them]...it would be important to have all of that information for someone as a health professional that could go back in and see your history. They're going to need more than just a brief description like the patient would want...you could offer both.... You know, this section is mostly for the patient to understand what they're looking at, and then this is the test results and the exact information that we based this information off of.

Participant #47

#### Design Recommendation 3: Ensure That Both Web and Smartphone Functionalities Are Accounted for in the Design

Many participants preferred to use their smartphones to manage their health and health care; however, several felt that patient portals are still designed for optimal use on a computer using a web-based layout rather than the more modular smartphone layout. As several participants pointed out, their phones were always with them, and they could use smartphones to share data in real time during clinical appointments, particularly when seeking care at a new or out-of-network clinic. As one participant described:

I had to go to the ER.... My home base is [institution name], but I went to [another place] this particular day because it was closer. Even though they are connected, they could not see my history. So rather than wait for my doctor, I just pulled up my history on my phone so the ER doctor could help diagnose me better. So even though he didn't get to speak to my doctor, he still had the reference and the notes to get the answers he needed. So even though the context didn't make sense to me, it made sense to him.

Participant #36

Several participants however expressed a concern that smartphone patient portal apps did not have the same range of functionality as that supported by computer-based applications, raising some points about the strengths and weaknesses of smartphone delivery versus computer delivery. Several commented on the size of smartphones being an inherent weakness when a lot of information or text needs to be displayed. These weaknesses have implications for designing usable results sections that meet the needs of patients and their health care providers. For instance, one participant said:

There's a lot in information on the page. It's a lot easier to see it all spread out on the computer...I think that was the problem on the smartphone. It was just hard to read.

Participant #8

### Domain 2: Content of the Patient-Friendly Results Summary Coversheet

#### Content Recommendation 1: Include a Personalized Note From the Clinician With the Electronic Test Results

For results that were returned via the internet, many participants felt that those results should include a personal message from a clinician. For some participants, the inclusion of such a personal note was the one thing that distinguished the web-based return of genetic results from the web-based return of routine test results (eg, blood panels):

...with the genetic testing [my clinician] did include the note right then. They normally don't do the note. So that was the difference, really, between the two, versus regular blood work results and genetic testing...she had a little note in there saying it was all clear...it made it a more personal experience, which I like a little better...as personal as you can get through an email or [a patient] portal site.

Participant #23

These personal notes helped to humanize the interactions for some participants, making them feel heard and improving their satisfaction with their care. One participant said:

Having that small note says that somebody is identifying that this is a real person...conversation, even if it is through email, or through [the patient portal]. It's still something, rather than pushing you through and here's your numbers, and if you have any questions, yeah, yeah, we'll call you. Or you can call us, but we're not going to call you.... Start with the note, and then, you can go to the test results…I feel way more informed, and more like everything is being taken care of. That I'm not being ignored.

Participant #51

#### Content Recommendation 2: Report Only Key information in the Results Summary Coversheet

Several participants mentioned that there was too much information in the report that they did not understand. As one participant succinctly put it:

You're overwhelmed by all of this jargon and underwhelmed at the same time by how little is actually said without directly telling you yes or no.

Participant #34

Another participant suggested that starting the report with an easily identifiable and comprehensible “bottom line” would be helpful:

We have this thing in the Navy, I don’t really know how it would be operationalized in healthcare, but we have this thing called BLUF.... When you're writing somebody an email...at the top of the email in capital letters you put B-L-U-F: stands for bottom line up front.... Okay, bottom line up front: “Of the 60 types of cancers we screened for, you are not genetically predisposed to any.” Then, “[Participant name], we did this test and…this is what it tested for, and while this is only looking at your genetic makeup and not looking at environmental factors…we estimate that you have this percentage chance....” The same thing is true whether it's the genetic testing or testing my lipids...particularly when you're talking about healthcare and something as complicated or convoluted as genetic testing, it would really seem to me that the person who is delivering the news either in writing, or over the telephone, or in person, needs the BLUF.

Participant #45

The extensive details in reports seemed unnecessary to participants who were largely focused on what the results would mean for them personally. For example, a participant said:

As a whole, the detail that they gave, I didn't understand. The end result, I understood. It was as clear as day because it [was] negative. When I look at the test results, they give me the gene sequence and value notes. They give me all this gene coding stuff…I don't know those from Adam.... But then it gives me the result, and it says negative for mutations, the interpretation. And...the disclosure statement saying, “Hey, even though this is what's found, we're not guaranteeing you anything....” The important parts are in bold, and I understood them just fine. The gene sequence: probably not all that important. Because for me, it doesn't do anything.

Participant #37

#### Content Recommendation 3: Use Clear, Accessible, Jargon-Free Language in the Results Summary Coversheet

Participants pointed out the disconnect between medical terms and how everyday people use language. Several participants suggested that the terms *positive* and *negative* were confusing in the context of how these words are used in the vernacular:

I feel like the positive and negative, it really trips me up. Like getting a...“Your HIV test came back negative.” And you're like, “Wait a minute. It's negative. Negative is good.” So, I think the negative and the positive, they're obviously not opposite meaning. It's very clear, like, ”This came back with nothing.“ Or ”This came back with....“ But it can be an immediate gut-wrenching reaction, of like, ”Uh-oh.“ I'm used to associating that word with a bad thing.

Participant #8

Using these words was particularly confusing when they were unaccompanied by an explanation or sufficient context. One participant stated:

...without getting into...too much detail. We were looking for [a] particular marker, right?...And in this case it was negative...And it tells me it's negative. But I don't have any of the qualitative information. Is negative good or bad?...And what’s it mean? ...it's not like something that you could just say, ”In range or out of range.“ Right? Is that thumbs up or thumbs down?

Participant #1

Some participants offered specific suggestions for sections in the coversheet where wording could be made friendlier for nonexperts and enhanced to provide reassurance:

...if it just says heterozygous they really don't know what that means...just put a sentence in there added to it saying, ”There's two copies. If you only have one copy, it's much less serious than if you have two copies.“ You know, ”If you do have two copies, we still have treatments that work,“ just like Dr. [provider name] explained to me. That little, short two-sentence explanation, would ease people’s minds.

Participant #40

#### Content Recommendation 4: Include Next Steps in the Results Summary Coversheet

Participants wanted to have major next steps included in the patient-friendly summary:

Definitely having some sort of ”so here are the recommended things that you should do“ so you can make educated choices about what you want to do now that you have those [risk] results…you might think, “Oh I have no chance of getting it.” That's not really true…[Negative result] might be a false sense of security.

Participant #58

In cases where next steps could not be included (eg, because they were complex or very individualized), participants wanted to know that next steps were coming in a more detailed follow-up, such as a conversation:

[There should be] a notice that the next steps were coming.... ”Based on our testing, you are predisposed to 14 different kinds of cancer. Action items: we need to meet to discuss this....“ ...follow up or action items, that is again, simple, declarative...and stands out visually.

Participant #45

[The genetic test results] didn't have any like, these are the health implications you could deal with for the rest of your life, or something like that. Or this is what you could possibly be dealing with. That was nonexistent...I would find it helpful to say like, maybe we schedule a follow-up visit if it warrants it, or maybe just a more detailed response on their part.

Participant #47

#### Content Recommendation 5: Include Encouragement and Easy-to-Find Information for Contacting the Health Care Provider if There Are Follow-up Questions

Several participants understood that complex results would likely be returned electronically in the future. As such, they believed that follow-up contact with a clinician was an extremely important service that should be accommodated. In the electronic report, participants wanted to receive both encouragement to follow up (ie, as a way to reinforce the fact that their potential concerns would be taken seriously) and the contact information of an appropriate health care provider:

I think maybe always giving the option of a follow-up and a personal note. Always include, ”If you would like to discuss more, feel free to call us” at this [number].“

Participant #23

Even when receiving written encouragement in the patient portal, some participants shared concerns that they would not feel comfortable with reaching out to busy health care providers with their questions. In this case, they preferred having a health care provider or a health care provider’s office contact them via a brief telephone call, rather than a note, to encourage them to ask any questions. One participant said:

I...think it's important to break the ice...even if eventually you get most of your results electronically, I still think it's important to have somebody, even if it's some sort of medical assistant in the office, call and say ”We're here for you and if you have any questions, please call us....“ Because I often hesitate because I think if they didn't say anything, and maybe I'm just stupid.

Participant #48

## Discussion

### Principal Results

Our study offers an exploration of both the design and content of electronic returns of genetic test results, sharing perspectives from adult patients who vary widely in terms of age; come from a large, urban, academic medical center; have undergone genetic testing; and are fluent in English. Results that are returned electronically should start with a summary coversheet containing the most pertinent information. For example, participants recommended that genetic test results should include simple summaries that provide an overview of their test results in an accessible language. This content would be placed at the beginning of the test results (eg, on the test result landing page for a specific result) and would function as a coversheet that precedes the more detailed clinical report. Many participants wanted a personal note from a clinician, and some participants suggested that this note should be placed at the very beginning of the electronic report. Participants offered specific feedback on content for the summary, which at a minimum should include the “bottom line” (eg, whether a medically important genetic variant was found), patients’ next steps, and explicit encouragement to contact health care providers with any questions or concerns. Summaries must be written in a clear language and avoid technical jargon, which might include avoiding the words *positive* and *negative* in this section. Importantly, participants wanted this summary coversheet in addition to—not in place of—the more detailed clinical report.

Many participants used both their computers and their smartphones to access their patient portals but found that while using a smartphone was very helpful, the interface was not optimal. As more patients across many demographics use smartphones to manage their health, it is important to prioritize designing genetic test results information for delivery on smartphones instead of test results that are more akin to genetic counseling results letters.

### Comparison With Prior Work

#### Attention to the Design of Electronic Delivery is Needed, as Genetic Tests Outpace Clinician Hours

Although there has been effort for designing letters to return genetic test results to patients [36], the specific challenges of leveraging web-based electronic capabilities for result returns have not yet been well explored [4]. Some of this delay might be due to service delivery models that mandate or strongly recommend in-person returns for test results that are deemed sensitive (eg, genetic [37,38] or radiology results [39,40]). Although the number of clinical genetic tests is on the rise, the supply of genetic counselors and other health care providers who are qualified to fully return results is not keeping apace [41-43]. Electronic portals may offer a patient-friendly and acceptable alternative for returning results that allows for the prioritization of genetic counselors’ time to address the most complex or sensitive genetic results [22]. Electronic portals have substantially more functionality than a simple paper letter; therefore, there is great potential for leveraging informational hierarchies, external links to additional information, and the patient-directed use of the result page for both patients’ own use and their physicians’ use. However, our data demonstrate that current approaches to electronic result conveyance do not meet patient needs, supporting the necessity for bringing attention to these design elements to make effective and acceptable use of this model, such as creating a summary coversheet template that has been user-tested with patients from a range of heath literacy and educational backgrounds.

#### Getting the Content Right Will Continue to Be a Critical Concern for the Electronic Return of Genetic Results

Genetic information has been described as “informationally complex” and “hard to interpret” even among medically trained professionals [44-47]. Similarly, our data show that some patients struggle to understand genetic results reports as they are currently written due to the volume of information to sift through; the use of medical terms; and the lack of straight-forward, lay-friendly interpretations of the results. Indeed, many genetic results letters still do not meet Centers for Disease Control and Prevention–recommended literacy levels for health-related communication [48,49], and as our English-fluent participants noted, results letters can be confusing when they convey informationally complex results or even fairly simple results, such as “positive” or “negative.” Specifically, the differences in the significance of various types of genetic results and these differences’ impact on returning genetic results to individuals have been discussed in several contexts [37]. Patient preferences for the return of negative test results generally exhibit more openness for impersonal returns (eg, via secure messaging) than that for returns of results that are not normal; however, patient preferences vary greatly, and as noted previously, negative genetic results may have nuanced implications [32,33]. Our data further support previous calls for the improved communication of genetic information to patients and the tailoring of these calls to the electronic return process.

#### User-Centered Approaches Are Needed When Developing Electronic Test Results Templates

Research with patients supports a user-centered design approach for the return of test results [35], including the return of genetic results [50]. Ensuring that portals are tailored to meet patient needs has the potential to not only ensure the appropriate delivery of results but also enable the use of patient portals to encourage appropriate follow-ups [22,51].

Many patient portals are add-ons to commercial EHR software packages; often, they are designed without patient or clinician input [4,52,53]. Ensuring that patient portals are able to deliver results on a range of electronic devices in ways that are user-centered, in terms of both design and content, is crucial [54].

We acknowledge that a patient-centered approach may elicit suggestions for content and design that might not be easily accommodated by available patient portal software (such as those available through EHR software), the clinical workflow of health care systems, or the preferences of individual health care providers. These issues are beyond the scope of our study but must be considered in the final decisions regarding the portal-based return of genetic results.

### Limitations

Focusing our study sample on the patient population of a single, although large, urban academic health system in the Pacific Northwest may have limited the scope of the views shared in this paper. Our participant cohort was largely female and White, and all participants were fluent English speakers who have used the patient portal. It is possible that underrepresented groups may tend to be nonusers of the patient portal [29,55] or tend to not undergo genetic testing [56]. Further, enrolled participants responded to email or phone invitations to participate in the study, which may have also biased our sample toward people who are more comfortable in engaging with research or medical concepts and thus may have a higher comfort level with receiving medical information via the internet than those who did not accept the invitations. All interviews were conducted by phone and in English; thus, our findings do not take into account views of people with limited English proficiency or those who are unable to use phones or other technology. Understanding the content needs of those with limited English proficiency is a crucial step toward ensuring that the development of patient portal services for result returns is appropriate for a wide range of users. Finally, as this was an exploratory qualitative study, we cannot estimate how widely shared our participants’ views are or whether they would be shared by patients in other geographic regions or health care systems. We also discovered through our qualitative interviews with patients that using patient portals to return certain results (eg, those that are considered particularly complex or fraught) should only be supplemental, as a conversation is usually preferred in such instances. Our paper on the types of tests should or could be returned electronically is forthcoming. This has limited what we have chosen to report with regard to patient recommendations for the content and design of returns of less fraught, but potentially confusing, genetic test results.

### Conclusions

Although research has been conducted to explore the needs of patients when genetic test results or other test results are returned and to determine some patient portal design needs, the design of electronic results reports lags behind patient consumers’ expectations for using and accessing their test results. Our study results indicate that patients value the details that are included in formal laboratory reports, but as many of them access their tests electronically through patient portals, including via their smartphones, report templates must take into consideration where, when, why, and how patients use their electronically available health information. Our participants recommended the creation of a coversheet that includes a brief “bottom line,” is easily accessible and visually distinct, and uses broadly understandable content that prioritizes next steps and encourages patients to follow up with their health care providers to obtain more information. It is important for this coversheet to be available in a usable form on smartphones, since many participants accessed their results and shared content (eg, with their clinicians during medical appointments) via their smartphones. There is a real opportunity for development approaches that use interaction design principles and user-centeredness in new ways beyond merely translating a detailed clinical report for electronic delivery.

## Abbreviations

EHR: electronic health record
UW: University of Washington
